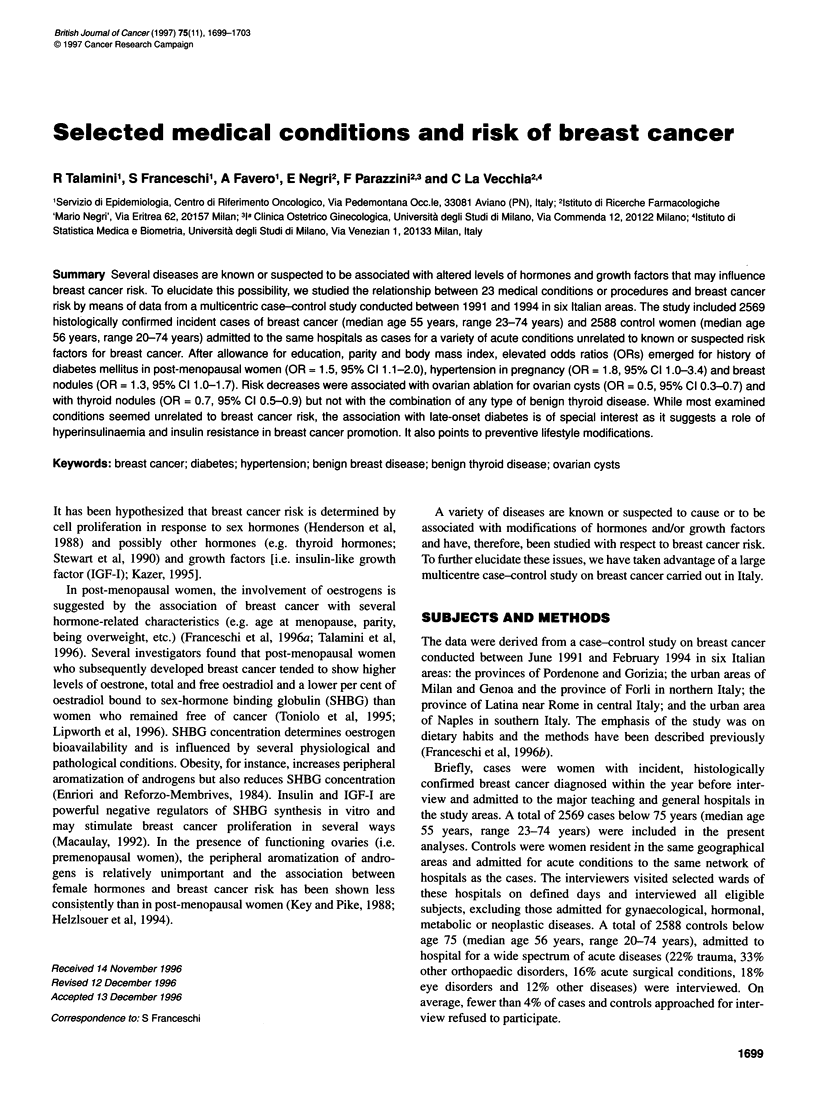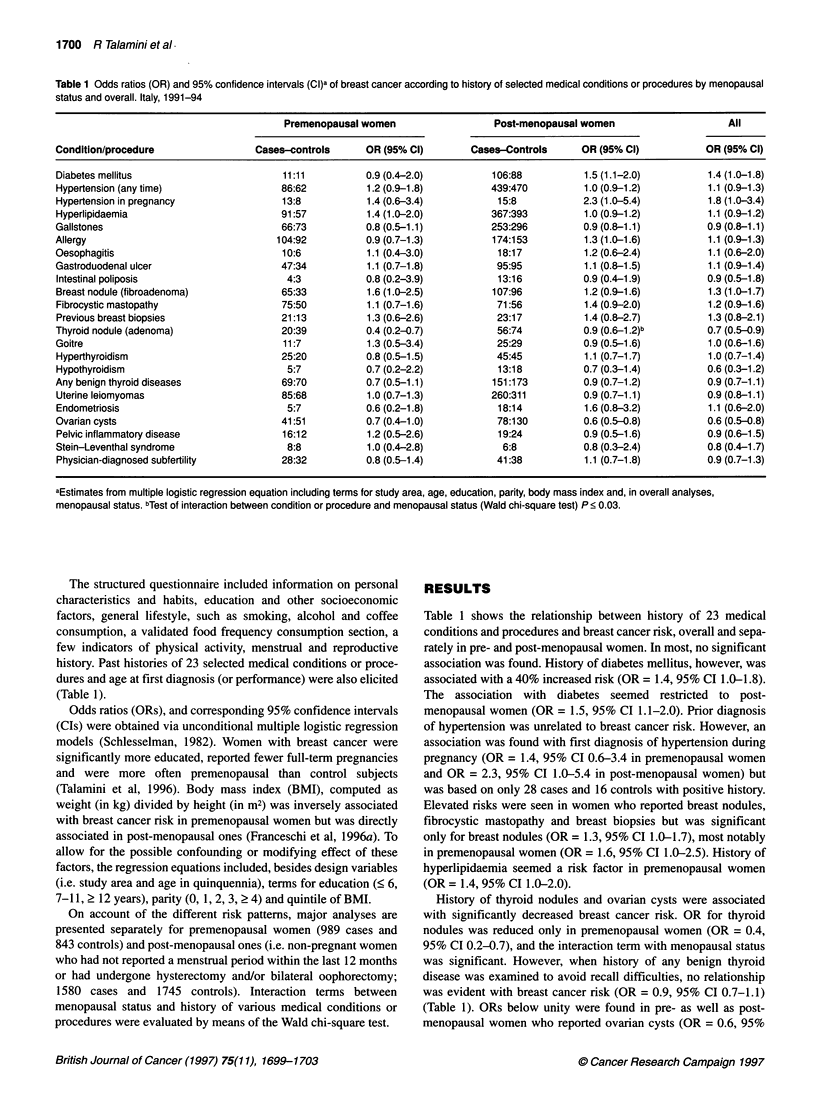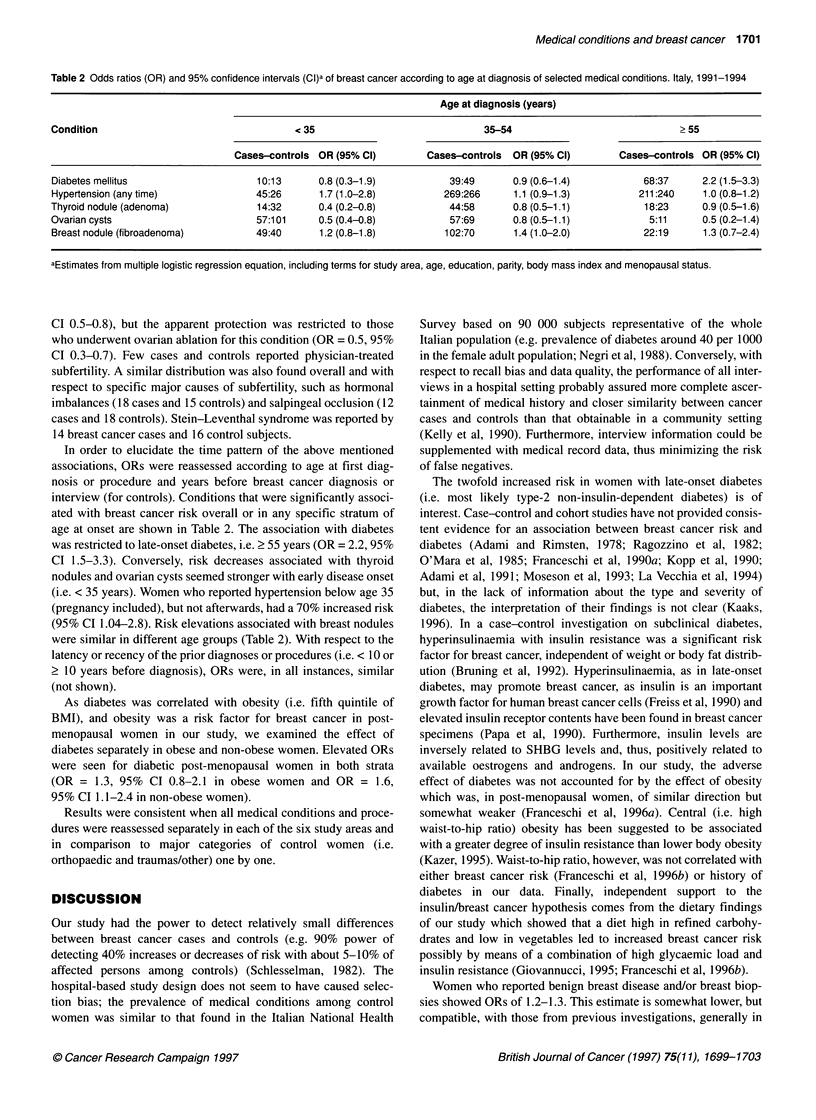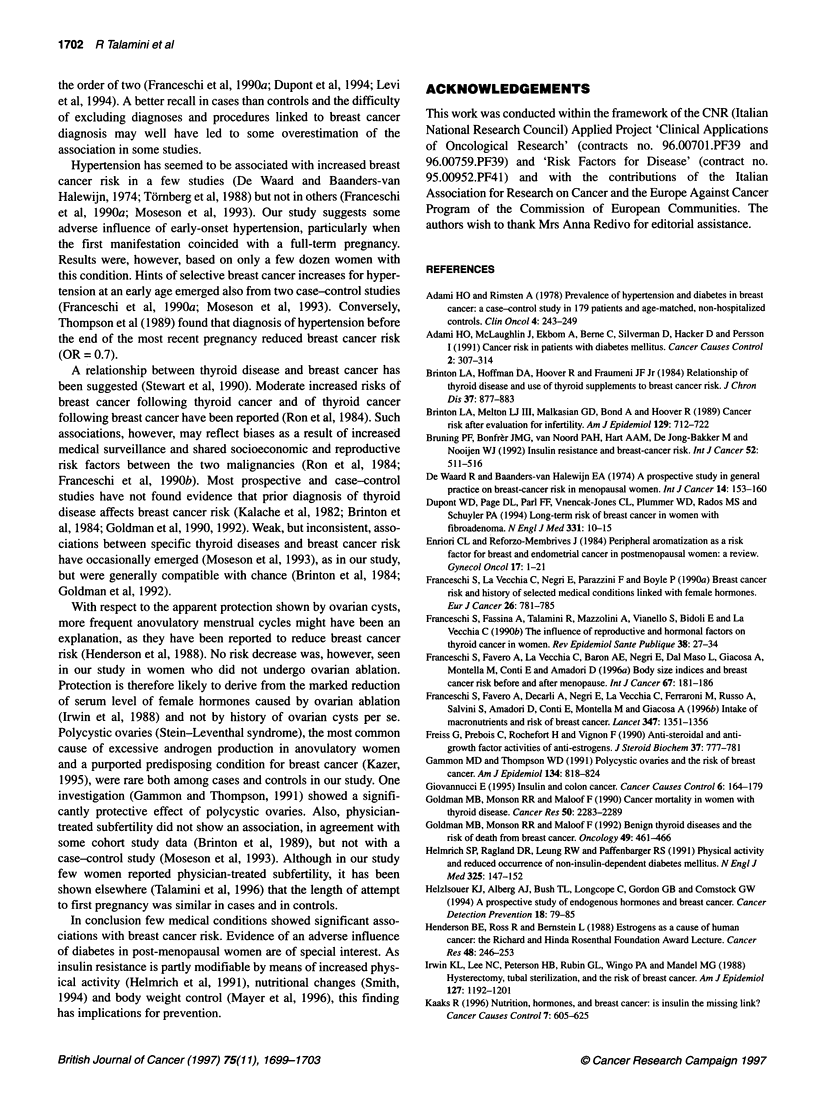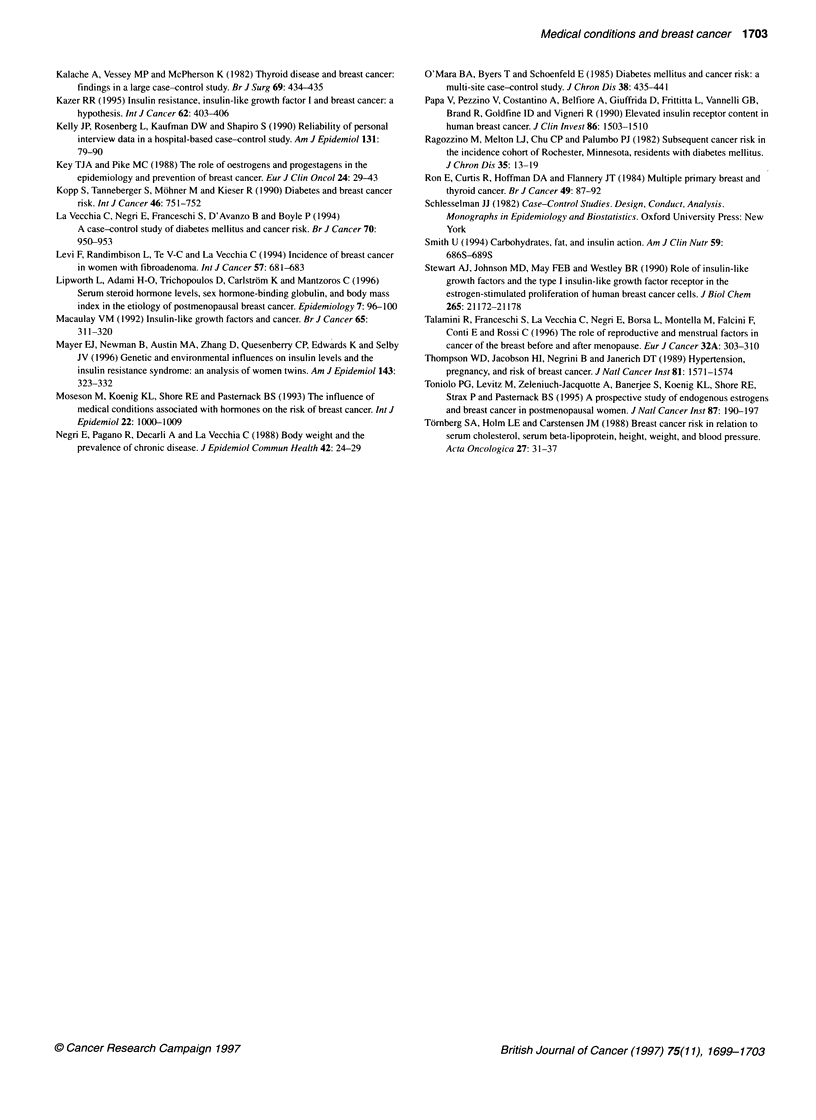# Selected medical conditions and risk of breast cancer.

**DOI:** 10.1038/bjc.1997.289

**Published:** 1997

**Authors:** R. Talamini, S. Franceschi, A. Favero, E. Negri, F. Parazzini, C. La Vecchia

**Affiliations:** Servizio di Epidemiologia, Centro di Riferimento Oncologico, Aviano (PN), Italy.

## Abstract

Several diseases are known or suspected to be associated with altered levels of hormones and growth factors that may influence breast cancer risk. To elucidate this possibility, we studied the relationship between 23 medical conditions or procedures and breast cancer risk by means of data from a multicentric case-control study conducted between 1991 and 1994 in six Italian areas. The study included 2569 histologically confirmed incident cases of breast cancer (median age 55 years, range 23-74 years) and 2588 control women (median age 56 years, range 20-74 years) admitted to the same hospitals as cases for a variety of acute conditions unrelated to known or suspected risk factors for breast cancer. After allowance for education, parity and body mass index, elevated odds ratios (ORs) emerged for history of diabetes mellitus in post-menopausal women (OR = 1.5, 95% CI 1.1-2.0), hypertension in pregnancy (OR = 1.8, 95% CI 1.0-3.4) and breast nodules (OR = 1.3, 95% CI 1.0-1.7). Risk decreases were associated with ovarian ablation for ovarian cysts (OR = 0.5, 95% CI 0.3-0.7) and with thyroid nodules (OR = 0.7, 95% CI 0.5-0.9) but not with the combination of any type of benign thyroid disease. While most examined conditions seemed unrelated to breast cancer risk, the association with late-onset diabetes is of special interest as it suggests a role of hyperinsulinaemia and insulin resistance in breast cancer promotion. It also points to preventive lifestyle modifications.


British Journal of Cancer (1997) 75(11), 1699–1703
© 1997 Cancer Research Campaign

### Selected medical conditions and risk of breast cancer

**R Talamini[1], S Franceschi[1], A Favero[1], E Negri[2], F Parazzini[2,3] and C La Vecchia[2,4]**

[1]Servizio di Epidemiologia, Centro di Riferimento Oncologico, Via Pedemontana Occ.le, 33081 Aviano (PN), Italy; [2]Istituto di Ricerche Farmacologiche 'Mario Negri', Via Eritrea 62, 20157 Milan; [3]Iª Clinica Ostetrico Ginecologica, Università degli Studi di Milano, Via Commenda 12, 20122 Milano; [4]Istituto di Statistica Medica e Biometria, Università degli Studi di Milano, Via Venezian 1, 20133 Milan, Italy

**Summary** Several diseases are known or suspected to be associated with altered levels of hormones and growth factors that may influence breast cancer risk. To elucidate this possibility, we studied the relationship between 23 medical conditions or procedures and breast cancer risk by means of data from a multicentric case–control study conducted between 1991 and 1994 in six Italian areas. The study included 2569 histologically confirmed incident cases of breast cancer (median age 55 years, range 23–74 years) and 2588 control women (median age 56 years, range 20–74 years) admitted to the same hospitals as cases for a variety of acute conditions unrelated to known or suspected risk factors for breast cancer. After allowance for education, parity and body mass index, elevated odds ratios (ORs) emerged for history of diabetes mellitus in post-menopausal women (OR = 1.5, 95% CI 1.1–2.0), hypertension in pregnancy (OR = 1.8, 95% CI 1.0–3.4) and breast nodules (OR = 1.3, 95% CI 1.0–1.7). Risk decreases were associated with ovarian ablation for ovarian cysts (OR = 0.5, 95% CI 0.3–0.7) and with thyroid nodules (OR = 0.7, 95% CI 0.5–0.9) but not with the combination of any type of benign thyroid disease. While most examined conditions seemed unrelated to breast cancer risk, the association with late-onset diabetes is of special interest as it suggests a role of hyperinsulinaemia and insulin resistance in breast cancer promotion. It also points to preventive lifestyle modifications.

**Keywords:** breast cancer; diabetes; hypertension; benign breast disease; benign thyroid disease; ovarian cysts

It has been hypothesized that breast cancer risk is determined by cell proliferation in response to sex hormones (Henderson et al, 1988) and possibly other hormones (e.g. thyroid hormones; Stewart et al, 1990) and growth factors [i.e. insulin-like growth factor (IGF-I); Kazer, 1995].

In post-menopausal women, the involvement of oestrogens is suggested by the association of breast cancer with several hormone-related characteristics (e.g. age at menopause, parity, being overweight, etc.) (Franceschi et al, 1996*a*; Talamini et al, 1996). Several investigators found that post-menopausal women who subsequently developed breast cancer tended to show higher levels of oestrone, total and free oestradiol and a lower per cent of oestradiol bound to sex-hormone binding globulin (SHBG) than women who remained free of cancer (Toniolo et al, 1995; Lipworth et al, 1996). SHBG concentration determines oestrogen bioavailability and is influenced by several physiological and pathological conditions. Obesity, for instance, increases peripheral aromatization of androgens but also reduces SHBG concentration (Enriori and Reforzo-Membrives, 1984). Insulin and IGF-I are powerful negative regulators of SHBG synthesis in vitro and may stimulate breast cancer proliferation in several ways (Macaulay, 1992). In the presence of functioning ovaries (i.e. premenopausal women), the peripheral aromatization of androgens is relatively unimportant and the association between female hormones and breast cancer risk has been shown less consistently than in post-menopausal women (Key and Pike, 1988; Helzlsouer et al, 1994).

*Received 14 November 1996*
*Revised 12 December 1996*
*Accepted 13 December 1996*

*Correspondence to:* S Franceschi

A variety of diseases are known or suspected to cause or to be associated with modifications of hormones and/or growth factors and have, therefore, been studied with respect to breast cancer risk. To further elucidate these issues, we have taken advantage of a large multicentre case–control study on breast cancer carried out in Italy.

### SUBJECTS AND METHODS

The data were derived from a case–control study on breast cancer conducted between June 1991 and February 1994 in six Italian areas: the provinces of Pordenone and Gorizia; the urban areas of Milan and Genoa and the province of Forli in northern Italy; the province of Latina near Rome in central Italy; and the urban area of Naples in southern Italy. The emphasis of the study was on dietary habits and the methods have been described previously (Franceschi et al, 1996*b*).

Briefly, cases were women with incident, histologically confirmed breast cancer diagnosed within the year before interview and admitted to the major teaching and general hospitals in the study areas. A total of 2569 cases below 75 years (median age 55 years, range 23–74 years) were included in the present analyses. Controls were women resident in the same geographical areas and admitted for acute conditions to the same network of hospitals as the cases. The interviewers visited selected wards of these hospitals on defined days and interviewed all eligible subjects, excluding those admitted for gynaecological, hormonal, metabolic or neoplastic diseases. A total of 2588 controls below age 75 (median age 56 years, range 20–74 years), admitted to hospital for a wide spectrum of acute diseases (22% trauma, 33% other orthopaedic disorders, 16% acute surgical conditions, 18% eye disorders and 12% other diseases) were interviewed. On average, fewer than 4% of cases and controls approached for interview refused to participate.

Table 1 Odds ratios (OR) and 95% confidence intervals (CI)[a] of breast cancer according to history of selected medical conditions or procedures by menopausal status and overall. Italy, 1991–94

| | Premenopausal women | | Post-menopausal women | | All |
|---|---|---|---|---|---|
| Condition/procedure | Cases–controls | OR (95% CI) | Cases–Controls | OR (95% CI) | OR (95% CI) |
| Diabetes mellitus | 11:11 | 0.9 (0.4–2.0) | 106:88 | 1.5 (1.1–2.0) | 1.4 (1.0–1.8) |
| Hypertension (any time) | 86:62 | 1.2 (0.9–1.8) | 439:470 | 1.0 (0.9–1.2) | 1.1 (0.9–1.3) |
| Hypertension in pregnancy | 13:8 | 1.4 (0.6–3.4) | 15:8 | 2.3 (1.0–5.4) | 1.8 (1.0–3.4) |
| Hyperlipidaemia | 91:57 | 1.4 (1.0–2.0) | 367:393 | 1.0 (0.9–1.2) | 1.1 (0.9–1.2) |
| Gallstones | 66:73 | 0.8 (0.5–1.1) | 253:296 | 0.9 (0.8–1.1) | 0.9 (0.8–1.1) |
| Allergy | 104:92 | 0.9 (0.7–1.3) | 174:153 | 1.3 (1.0–1.6) | 1.1 (0.9–1.3) |
| Oesophagitis | 10:6 | 1.1 (0.4–3.0) | 18:17 | 1.2 (0.6–2.4) | 1.1 (0.6–2.0) |
| Gastroduodenal ulcer | 47:34 | 1.1 (0.7–1.8) | 95:95 | 1.1 (0.8–1.5) | 1.1 (0.9–1.4) |
| Intestinal poliposis | 4:3 | 0.8 (0.2–3.9) | 13:16 | 0.9 (0.4–1.9) | 0.9 (0.5–1.8) |
| Breast nodule (fibroadenoma) | 65:33 | 1.6 (1.0–2.5) | 107:96 | 1.2 (0.9–1.6) | 1.3 (1.0–1.7) |
| Fibrocystic mastopathy | 75:50 | 1.1 (0.7–1.6) | 71:56 | 1.4 (0.9–2.0) | 1.2 (0.9–1.6) |
| Previous breast biopsies | 21:13 | 1.3 (0.6–2.6) | 23:17 | 1.4 (0.8–2.7) | 1.3 (0.8–2.1) |
| Thyroid nodule (adenoma) | 20:39 | 0.4 (0.2–0.7) | 56:74 | 0.9 (0.6–1.2)[b] | 0.7 (0.5–0.9) |
| Goitre | 11:7 | 1.3 (0.5–3.4) | 25:29 | 0.9 (0.5–1.6) | 1.0 (0.6–1.6) |
| Hyperthyroidism | 25:20 | 0.8 (0.5–1.5) | 45:45 | 1.1 (0.7–1.7) | 1.0 (0.7–1.4) |
| Hypothyroidism | 5:7 | 0.7 (0.2–2.2) | 13:18 | 0.7 (0.3–1.4) | 0.6 (0.3–1.2) |
| Any benign thyroid diseases | 69:70 | 0.7 (0.5–1.1) | 151:173 | 0.9 (0.7–1.2) | 0.9 (0.7–1.1) |
| Uterine leiomyomas | 85:68 | 1.0 (0.7–1.3) | 260:311 | 0.9 (0.7–1.1) | 0.9 (0.8–1.1) |
| Endometriosis | 5:7 | 0.6 (0.2–1.8) | 18:14 | 1.6 (0.8–3.2) | 1.1 (0.6–2.0) |
| Ovarian cysts | 41:51 | 0.7 (0.4–1.0) | 78:130 | 0.6 (0.5–0.8) | 0.6 (0.5–0.8) |
| Pelvic inflammatory disease | 16:12 | 1.2 (0.5–2.6) | 19:24 | 0.9 (0.5–1.6) | 0.9 (0.6–1.5) |
| Stein–Leventhal syndrome | 8:8 | 1.0 (0.4–2.8) | 6:8 | 0.8 (0.3–2.4) | 0.8 (0.4–1.7) |
| Physician-diagnosed subfertility | 28:32 | 0.8 (0.5–1.4) | 41:38 | 1.1 (0.7–1.8) | 0.9 (0.7–1.3) |

[a]Estimates from multiple logistic regression equation including terms for study area, age, education, parity, body mass index and, in overall analyses, menopausal status. [b]Test of interaction between condition or procedure and menopausal status (Wald chi-square test) $P \leq 0.03$.

The structured questionnaire included information on personal characteristics and habits, education and other socioeconomic factors, general lifestyle, such as smoking, alcohol and coffee consumption, a validated food frequency consumption section, a few indicators of physical activity, menstrual and reproductive history. Past histories of 23 selected medical conditions or procedures and age at first diagnosis (or performance) were also elicited (Table 1).

Odds ratios (ORs), and corresponding 95% confidence intervals (CIs) were obtained via unconditional multiple logistic regression models (Schlesselman, 1982). Women with breast cancer were significantly more educated, reported fewer full-term pregnancies and were more often premenopausal than control subjects (Talamini et al, 1996). Body mass index (BMI), computed as weight (in kg) divided by height (in $m^2$) was inversely associated with breast cancer risk in premenopausal women but was directly associated in post-menopausal ones (Franceschi et al, 1996*a*). To allow for the possible confounding or modifying effect of these factors, the regression equations included, besides design variables (i.e. study area and age in quinquennia), terms for education (≤ 6, 7–11, ≥ 12 years), parity (0, 1, 2, 3, ≥ 4) and quintile of BMI.

On account of the different risk patterns, major analyses are presented separately for premenopausal women (989 cases and 843 controls) and post-menopausal ones (i.e. non-pregnant women who had not reported a menstrual period within the last 12 months or had undergone hysterectomy and/or bilateral oophorectomy; 1580 cases and 1745 controls). Interaction terms between menopausal status and history of various medical conditions or procedures were evaluated by means of the Wald chi-square test.

### RESULTS

Table 1 shows the relationship between history of 23 medical conditions and procedures and breast cancer risk, overall and separately in pre- and post-menopausal women. In most, no significant association was found. History of diabetes mellitus, however, was associated with a 40% increased risk (OR = 1.4, 95% CI 1.0–1.8). The association with diabetes seemed restricted to post-menopausal women (OR = 1.5, 95% CI 1.1–2.0). Prior diagnosis of hypertension was unrelated to breast cancer risk. However, an association was found with first diagnosis of hypertension during pregnancy (OR = 1.4, 95% CI 0.6–3.4 in premenopausal women and OR = 2.3, 95% CI 1.0–5.4 in post-menopausal women) but was based on only 28 cases and 16 controls with positive history. Elevated risks were seen in women who reported breast nodules, fibrocystic mastopathy and breast biopsies but was significant only for breast nodules (OR = 1.3, 95% CI 1.0–1.7), most notably in premenopausal women (OR = 1.6, 95% CI 1.0–2.5). History of hyperlipidaemia seemed a risk factor in premenopausal women (OR = 1.4, 95% CI 1.0–2.0).

History of thyroid nodules and ovarian cysts were associated with significantly decreased breast cancer risk. OR for thyroid nodules was reduced only in premenopausal women (OR = 0.4, 95% CI 0.2–0.7), and the interaction term with menopausal status was significant. However, when history of any benign thyroid disease was examined to avoid recall difficulties, no relationship was evident with breast cancer risk (OR = 0.9, 95% CI 0.7–1.1) (Table 1). ORs below unity were found in pre- as well as post-menopausal women who reported ovarian cysts (OR = 0.6, 95%

 © Cancer Research Campaign 1997

**Table 2** Odds ratios (OR) and 95% confidence intervals (CI)[a] of breast cancer according to age at diagnosis of selected medical conditions. Italy, 1991–1994

| Condition | Age at diagnosis (years) | | | | | |
|---|---|---|---|---|---|---|
| | < 35 | | 35–54 | | ≥ 55 | |
| | Cases–controls | OR (95% CI) | Cases–controls | OR (95% CI) | Cases–controls | OR (95% CI) |
| Diabetes mellitus | 10:13 | 0.8 (0.3–1.9) | 39:49 | 0.9 (0.6–1.4) | 68:37 | 2.2 (1.5–3.3) |
| Hypertension (any time) | 45:26 | 1.7 (1.0–2.8) | 269:266 | 1.1 (0.9–1.3) | 211:240 | 1.0 (0.8–1.2) |
| Thyroid nodule (adenoma) | 14:32 | 0.4 (0.2–0.8) | 44:58 | 0.8 (0.5–1.1) | 18:23 | 0.9 (0.5–1.6) |
| Ovarian cysts | 57:101 | 0.5 (0.4–0.8) | 57:69 | 0.8 (0.5–1.1) | 5:11 | 0.5 (0.2–1.4) |
| Breast nodule (fibroadenoma) | 49:40 | 1.2 (0.8–1.8) | 102:70 | 1.4 (1.0–2.0) | 22:19 | 1.3 (0.7–2.4) |

[a]Estimates from multiple logistic regression equation, including terms for study area, age, education, parity, body mass index and menopausal status.

CI 0.5–0.8), but the apparent protection was restricted to those who underwent ovarian ablation for this condition (OR = 0.5, 95% CI 0.3–0.7). Few cases and controls reported physician-treated subfertility. A similar distribution was also found overall and with respect to specific major causes of subfertility, such as hormonal imbalances (18 cases and 15 controls) and salpingeal occlusion (12 cases and 18 controls). Stein–Leventhal syndrome was reported by 14 breast cancer cases and 16 control subjects.

In order to elucidate the time pattern of the above mentioned associations, ORs were reassessed according to age at first diagnosis or procedure and years before breast cancer diagnosis or interview (for controls). Conditions that were significantly associated with breast cancer risk overall or in any specific stratum of age at onset are shown in Table 2. The association with diabetes was restricted to late-onset diabetes, i.e. ≥ 55 years (OR = 2.2, 95% CI 1.5–3.3). Conversely, risk decreases associated with thyroid nodules and ovarian cysts seemed stronger with early disease onset (i.e. < 35 years). Women who reported hypertension below age 35 (pregnancy included), but not afterwards, had a 70% increased risk (95% CI 1.04–2.8). Risk elevations associated with breast nodules were similar in different age groups (Table 2). With respect to the latency or recency of the prior diagnoses or procedures (i.e. < 10 or ≥ 10 years before diagnosis), ORs were, in all instances, similar (not shown).

As diabetes was correlated with obesity (i.e. fifth quintile of BMI), and obesity was a risk factor for breast cancer in post-menopausal women in our study, we examined the effect of diabetes separately in obese and non-obese women. Elevated ORs were seen for diabetic post-menopausal women in both strata (OR = 1.3, 95% CI 0.8–2.1 in obese women and OR = 1.6, 95% CI 1.1–2.4 in non-obese women).

Results were consistent when all medical conditions and procedures were reassessed separately in each of the six study areas and in comparison to major categories of control women (i.e. orthopaedic and traumas/other) one by one.

### DISCUSSION

Our study had the power to detect relatively small differences between breast cancer cases and controls (e.g. 90% power of detecting 40% increases or decreases of risk with about 5–10% of affected persons among controls) (Schlesselman, 1982). The hospital-based study design does not seem to have caused selection bias; the prevalence of medical conditions among control women was similar to that found in the Italian National Health Survey based on 90 000 subjects representative of the whole Italian population (e.g. prevalence of diabetes around 40 per 1000 in the female adult population; Negri et al, 1988). Conversely, with respect to recall bias and data quality, the performance of all interviews in a hospital setting probably assured more complete ascertainment of medical history and closer similarity between cancer cases and controls than that obtainable in a community setting (Kelly et al, 1990). Furthermore, interview information could be supplemented with medical record data, thus minimizing the risk of false negatives.

The twofold increased risk in women with late-onset diabetes (i.e. most likely type-2 non-insulin-dependent diabetes) is of interest. Case–control and cohort studies have not provided consistent evidence for an association between breast cancer risk and diabetes (Adami and Rimsten, 1978; Ragozzino et al, 1982; O'Mara et al, 1985; Franceschi et al, 1990*a*; Kopp et al, 1990; Adami et al, 1991; Moseson et al, 1993; La Vecchia et al, 1994) but, in the lack of information about the type and severity of diabetes, the interpretation of their findings is not clear (Kaaks, 1996). In a case–control investigation on subclinical diabetes, hyperinsulinaemia with insulin resistance was a significant risk factor for breast cancer, independent of weight or body fat distribution (Bruning et al, 1992). Hyperinsulinaemia, as in late-onset diabetes, may promote breast cancer, as insulin is an important growth factor for human breast cancer cells (Freiss et al, 1990) and elevated insulin receptor contents have been found in breast cancer specimens (Papa et al, 1990). Furthermore, insulin levels are inversely related to SHBG levels and, thus, positively related to available oestrogens and androgens. In our study, the adverse effect of diabetes was not accounted for by the effect of obesity which was, in post-menopausal women, of similar direction but somewhat weaker (Franceschi et al, 1996*a*). Central (i.e. high waist-to-hip ratio) obesity has been suggested to be associated with a greater degree of insulin resistance than lower body obesity (Kazer, 1995). Waist-to-hip ratio, however, was not correlated with either breast cancer risk (Franceschi et al, 1996*b*) or history of diabetes in our data. Finally, independent support to the insulin/breast cancer hypothesis comes from the dietary findings of our study which showed that a diet high in refined carbohydrates and low in vegetables led to increased breast cancer risk possibly by means of a combination of high glycaemic load and insulin resistance (Giovannucci, 1995; Franceschi et al, 1996*b*).

Women who reported benign breast disease and/or breast biopsies showed ORs of 1.2–1.3. This estimate is somewhat lower, but compatible, with those from previous investigations, generally in

© Cancer Research Campaign 1997

the order of two (Franceschi et al, 1990*a*; Dupont et al, 1994; Levi et al, 1994). A better recall in cases than controls and the difficulty of excluding diagnoses and procedures linked to breast cancer diagnosis may well have led to some overestimation of the association in some studies.

Hypertension has seemed to be associated with increased breast cancer risk in a few studies (De Waard and Baanders-van Halewijn, 1974; Törnberg et al, 1988) but not in others (Franceschi et al, 1990*a*; Moseson et al, 1993). Our study suggests some adverse influence of early-onset hypertension, particularly when the first manifestation coincided with a full-term pregnancy. Results were, however, based on only a few dozen women with this condition. Hints of selective breast cancer increases for hypertension at an early age emerged also from two case–control studies (Franceschi et al, 1990*a*; Moseson et al, 1993). Conversely, Thompson et al (1989) found that diagnosis of hypertension before the end of the most recent pregnancy reduced breast cancer risk (OR = 0.7).

A relationship between thyroid disease and breast cancer has been suggested (Stewart et al, 1990). Moderate increased risks of breast cancer following thyroid cancer and of thyroid cancer following breast cancer have been reported (Ron et al, 1984). Such associations, however, may reflect biases as a result of increased medical surveillance and shared socioeconomic and reproductive risk factors between the two malignancies (Ron et al, 1984; Franceschi et al, 1990*b*). Most prospective and case–control studies have not found evidence that prior diagnosis of thyroid disease affects breast cancer risk (Kalache et al, 1982; Brinton et al, 1984; Goldman et al, 1990, 1992). Weak, but inconsistent, associations between specific thyroid diseases and breast cancer risk have occasionally emerged (Moseson et al, 1993), as in our study, but were generally compatible with chance (Brinton et al, 1984; Goldman et al, 1992).

With respect to the apparent protection shown by ovarian cysts, more frequent anovulatory menstrual cycles might have been an explanation, as they have been reported to reduce breast cancer risk (Henderson et al, 1988). No risk decrease was, however, seen in our study in women who did not undergo ovarian ablation. Protection is therefore likely to derive from the marked reduction of serum level of female hormones caused by ovarian ablation (Irwin et al, 1988) and not by history of ovarian cysts per se. Polycystic ovaries (Stein–Leventhal syndrome), the most common cause of excessive androgen production in anovulatory women and a purported predisposing condition for breast cancer (Kazer, 1995), were rare both among cases and controls in our study. One investigation (Gammon and Thompson, 1991) showed a significantly protective effect of polycystic ovaries. Also, physician-treated subfertility did not show an association, in agreement with some cohort study data (Brinton et al, 1989), but not with a case–control study (Moseson et al, 1993). Although in our study few women reported physician-treated subfertility, it has been shown elsewhere (Talamini et al, 1996) that the length of attempt to first pregnancy was similar in cases and in controls.

In conclusion few medical conditions showed significant associations with breast cancer risk. Evidence of an adverse influence of diabetes in post-menopausal women are of special interest. As insulin resistance is partly modifiable by means of increased physical activity (Helmrich et al, 1991), nutritional changes (Smith, 1994) and body weight control (Mayer et al, 1996), this finding has implications for prevention.

### ACKNOWLEDGEMENTS

This work was conducted within the framework of the CNR (Italian National Research Council) Applied Project 'Clinical Applications of Oncological Research' (contracts no. 96.00701.PF39 and 96.00759.PF39) and 'Risk Factors for Disease' (contract no. 95.00952.PF41) and with the contributions of the Italian Association for Research on Cancer and the Europe Against Cancer Program of the Commission of European Communities. The authors wish to thank Mrs Anna Redivo for editorial assistance.

### REFERENCES

Adami HO and Rimsten A (1978) Prevalence of hypertension and diabetes in breast cancer: a case–control study in 179 patients and age-matched, non-hospitalized controls. *Clin Oncol* **4**: 243–249

Adami HO, McLaughlin J, Ekbom A, Berne C, Silverman D, Hacker D and Persson I (1991) Cancer risk in patients with diabetes mellitus. *Cancer Causes Control* **2**: 307–314

Brinton LA, Hoffman DA, Hoover R and Fraumeni JF Jr (1984) Relationship of thyroid disease and use of thyroid supplements to breast cancer risk. *J Chron Dis* **37**: 877–883

Brinton LA, Melton LJ III, Malkasian GD, Bond A and Hoover R (1989) Cancer risk after evaluation for infertility. *Am J Epidemiol* **129**: 712–722

Bruning PF, Bonfrèr JMG, van Noord PAH, Hart AAM, De Jong-Bakker M and Nooijen WJ (1992) Insulin resistance and breast-cancer risk. *Int J Cancer* **52**: 511–516

De Waard R and Baanders-van Halewijn EA (1974) A prospective study in general practice on breast-cancer risk in menopausal women. *Int J Cancer* **14**: 153–160

Dupont WD, Page DL, Parl FF, Vnencak-Jones CL, Plummer WD, Rados MS and Schuyler PA (1994) Long-term risk of breast cancer in women with fibroadenoma. *N Engl J Med* **331**: 10–15

Enriori CL and Reforzo-Membrives J (1984) Peripheral aromatization as a risk factor for breast and endometrial cancer in postmenopausal women: a review. *Gynecol Oncol* **17**: 1–21

Franceschi S, La Vecchia C, Negri E, Parazzini F and Boyle P (1990*a*) Breast cancer risk and history of selected medical conditions linked with female hormones. *Eur J Cancer* **26**: 781–785

Franceschi S, Fassina A, Talamini R, Mazzolini A, Vianello S, Bidoli E and La Vecchia C (1990*b*) The influence of reproductive and hormonal factors on thyroid cancer in women. *Rev Epidemiol Sante Publique* **38**: 27–34

Franceschi S, Favero A, La Vecchia C, Baron AE, Negri E, Dal Maso L, Giacosa A, Montella M, Conti E and Amadori D (1996*a*) Body size indices and breast cancer risk before and after menopause. *Int J Cancer* **67**: 181–186

Franceschi S, Favero A, Decarli A, Negri E, La Vecchia C, Ferraroni M, Russo A, Salvini S, Amadori D, Conti E, Montella M and Giacosa A (1996*b*) Intake of macronutrients and risk of breast cancer. *Lancet* **347**: 1351–1356

Freiss G, Prebois C, Rochefort H and Vignon F (1990) Anti-steroidal and anti-growth factor activities of anti-estrogens. *J Steroid Biochem* **37**: 777–781

Gammon MD and Thompson WD (1991) Polycystic ovaries and the risk of breast cancer. *Am J Epidemiol* **134**: 818–824

Giovannucci E (1995) Insulin and colon cancer. *Cancer Causes Control* **6**: 164–179

Goldman MB, Monson RR and Maloof F (1990) Cancer mortality in women with thyroid disease. *Cancer Res* **50**: 2283–2289

Goldman MB, Monson RR and Maloof F (1992) Benign thyroid diseases and the risk of death from breast cancer. *Oncology* **49**: 461–466

Helmrich SP, Ragland DR, Leung RW and Paffenbarger RS (1991) Physical activity and reduced occurrence of non-insulin-dependent diabetes mellitus. *N Engl J Med* **325**: 147–152

Helzlsouer KJ, Alberg AJ, Bush TL, Longcope C, Gordon GB and Comstock GW (1994) A prospective study of endogenous hormones and breast cancer. *Cancer Detection Prevention* **18**: 79–85

Henderson BE, Ross R and Bernstein L (1988) Estrogens as a cause of human cancer: the Richard and Hinda Rosenthal Foundation Award Lecture. *Cancer Res* **48**: 246–253

Irwin KL, Lee NC, Peterson HB, Rubin GL, Wingo PA and Mandel MG (1988) Hysterectomy, tubal sterilization, and the risk of breast cancer. *Am J Epidemiol* **127**: 1192–1201

Kaaks R (1996) Nutrition, hormones, and breast cancer: is insulin the missing link? *Cancer Causes Control* **7**: 605–625

© Cancer Research Campaign 1997

Kalache A, Vessey MP and McPherson K (1982) Thyroid disease and breast cancer: findings in a large case–control study. *Br J Surg* **69**: 434–435

Kazer RR (1995) Insulin resistance, insulin-like growth factor I and breast cancer: a hypothesis. *Int J Cancer* **62**: 403–406

Kelly JP, Rosenberg L, Kaufman DW and Shapiro S (1990) Reliability of personal interview data in a hospital-based case–control study. *Am J Epidemiol* **131**: 79–90

Key TJA and Pike MC (1988) The role of oestrogens and progestagens in the epidemiology and prevention of breast cancer. *Eur J Clin Oncol* **24**: 29–43

Kopp S, Tanneberger S, Möhner M and Kieser R (1990) Diabetes and breast cancer risk. *Int J Cancer* **46**: 751–752

La Vecchia C, Negri E, Franceschi S, D'Avanzo B and Boyle P (1994) A case–control study of diabetes mellitus and cancer risk. *Br J Cancer* **70**: 950–953

Levi F, Randimbison L, Te V-C and La Vecchia C (1994) Incidence of breast cancer in women with fibroadenoma. *Int J Cancer* **57**: 681–683

Lipworth L, Adami H-O, Trichopoulos D, Carlström K and Mantzoros C (1996) Serum steroid hormone levels, sex hormone-binding globulin, and body mass index in the etiology of postmenopausal breast cancer. *Epidemiology* **7**: 96–100

Macaulay VM (1992) Insulin-like growth factors and cancer. *Br J Cancer* **65**: 311–320

Mayer EJ, Newman B, Austin MA, Zhang D, Quesenberry CP, Edwards K and Selby JV (1996) Genetic and environmental influences on insulin levels and the insulin resistance syndrome: an analysis of women twins. *Am J Epidemiol* **143**: 323–332

Moseson M, Koenig KL, Shore RE and Pasternack BS (1993) The influence of medical conditions associated with hormones on the risk of breast cancer. *Int J Epidemiol* **22**: 1000–1009

Negri E, Pagano R, Decarli A and La Vecchia C (1988) Body weight and the prevalence of chronic disease. *J Epidemiol Commun Health* **42**: 24–29

O'Mara BA, Byers T and Schoenfeld E (1985) Diabetes mellitus and cancer risk: a multi-site case–control study. *J Chron Dis* **38**: 435–441

Papa V, Pezzino V, Costantino A, Belfiore A, Giuffrida D, Frittitta L, Vannelli GB, Brand R, Goldfine ID and Vigneri R (1990) Elevated insulin receptor content in human breast cancer. *J Clin Invest* **86**: 1503–1510

Ragozzino M, Melton LJ, Chu CP and Palumbo PJ (1982) Subsequent cancer risk in the incidence cohort of Rochester, Minnesota, residents with diabetes mellitus. *J Chron Dis* **35**: 13–19

Ron E, Curtis R, Hoffman DA and Flannery JT (1984) Multiple primary breast and thyroid cancer. *Br J Cancer* **49**: 87–92

Schlesselman JJ (1982) *Case–Control Studies. Design, Conduct, Analysis. Monographs in Epidemiology and Biostatistics.* Oxford University Press: New York

Smith U (1994) Carbohydrates, fat, and insulin action. *Am J Clin Nutr* **59**: 686S–689S

Stewart AJ, Johnson MD, May FEB and Westley BR (1990) Role of insulin-like growth factors and the type I insulin-like growth factor receptor in the estrogen-stimulated proliferation of human breast cancer cells. *J Biol Chem* **265**: 21172–21178

Talamini R, Franceschi S, La Vecchia C, Negri E, Borsa L, Montella M, Falcini F, Conti E and Rossi C (1996) The role of reproductive and menstrual factors in cancer of the breast before and after menopause. *Eur J Cancer* **32A**: 303–310

Thompson WD, Jacobson HI, Negrini B and Janerich DT (1989) Hypertension, pregnancy, and risk of breast cancer. *J Natl Cancer Inst* **81**: 1571–1574

Toniolo PG, Levitz M, Zeleniuch-Jacquotte A, Banerjee S, Koenig KL, Shore RE, Strax P and Pasternack BS (1995) A prospective study of endogenous estrogens and breast cancer in postmenopausal women. *J Natl Cancer Inst* **87**: 190–197

Törnberg SA, Holm LE and Carstensen JM (1988) Breast cancer risk in relation to serum cholesterol, serum beta-lipoprotein, height, weight, and blood pressure. *Acta Oncologica* **27**: 31–37

© Cancer Research Campaign 1997